# Impact de la pandémie de la COVID-19 sur l’utilisation des services de santé dans la ville de Niamey: une analyse dans 17 formations sanitaires de janvier à juin 2020

**DOI:** 10.11604/pamj.2021.39.159.28282

**Published:** 2021-07-01

**Authors:** Mariama Baissa Abdoulaye, Batouré Oumarou, Haladou Moussa, Blanche-Philomene Melanga Anya, Tambwe Didier, Biey Joseph Nsiari-muzeyi, Patrick Katoto, Charles Shey Wiysonge

**Affiliations:** 1 Country Office, World Health Organization, Quartier Plateau, Avenue Mohamed VI 1204, Niamey, Niger,; 2 Sub-regional Office for West Africa, World Health Organization, Independence street, Gate 0058, Ouagadougou, Burkina Faso,; 3 Centre for Infectious Diseases, Faculty of Medicine and Health Sciences, Stellenbosch University, Francie van Zijl Drive, Tygerberg 7505, Cape Town, South Africa,; 4 Centre for Tropical Medicine and Global Health, Faculty of Medicine, Catholic University of Bukavu, Bugabo 02, Bukavu, Democratic Republic of Congo,; 5 Department of Global Health, Faculty of Medicine and Health Sciences, Stellenbosch University, Francie van Zijl Drive, Tygerberg 7505, Cape Town, South Africa,; 6 Cochrane South Africa, South African Medical Research Council, Francie van Zijl Drive, Parow Valley 7501, Cape Town, South Africa,; 7 School of Public Health and Family Medicine, University of Cape Town, Anzio Road, Observatory 7935, Cape Town, South Africa

**Keywords:** Soins de santé, primaire, promotion de la santé, vaccination, SARS-COV-2, Niger, Primary health care, health promotion, immunization, SARS-CoV-2, Niger

## Abstract

Le défi que pose la pandémie de la COVID-19 sur le système de santé en Afrique est énorme mais pas bien quantifié en ce jour. Nous avons évalué les conséquences de la COVID-19 sur les activités curatives et préventives des formations sanitaires sur une période de six mois au niveau de 17 centres de sante intégré au Niamey de manière en comparant la première moitié de l´année 2020 à celle de l´année 2019. Les différences furent plus prononcées au deuxième trimestre 2020, avec une réduction de 34% (95%IC: -47% à -21%) pour les soins curatifs, 61% (95%IC: -74% à -48%) pour la vaccination aux pentavalents 1 et 3 et de 36% (95%IC: -49% à -23%) pour la VAR 1. Un gain quasi nul de 1% (95%IC: -2% à 4%) fut noté pour la fréquentation à la consultation prénatale annulant ainsi les acquis du premier trimestre. La pandémie de la COVID-19 impacte négativement sur les prestations de service destinées aux groupes les plus à risques de la population à savoir les femmes et les enfants. Des nouvelles stratégies comme l´engagement communautaire sont urgentes.

## Abstract (en)

COVID-19 pandemic has posed huge challenges for the health system in Africa; however they haven´t been well quantified. The purpose of this study was to assess the impact of COVID-19 pandemic on curative and preventive activities in health care facilities at 17 integrated health centers in Niamey by comparing the first half of 2020 and the first half of 2019. The differences were more pronounced in the second quarter of 2020, with a 34% reduction (95% CI: -47% to -21%) for curative care, 61% (95% CI: -74% to -48%) for pentavalent vaccines 1 and 3 and 36% (95% CI: -49% to -23%) for VAR 1. A nearly zero gain of 1% (95% IC: -2% to 4%) was reported for prenatal care attendance, thus reversing the gains of the first quarter. The COVID-19 pandemic has had negative effects on service deliveries to the most vulnerable groups, such as women and children. New strategies, such as community engagement, are essential.

## Introduction

La pandémie de l´infection à Coronavirus (COVID-19) est responsable d'une morbidité et mortalité très élevée dans le monde. Plusieurs pays en Afrique à l´instar du Niger, continuent d´enregistrer des cas d´infection due au SARS-CoV-2. A la date du 12 Novembre 2020, 1282 cas ont été diagnostiqués au Niger [1]. Au Niger, grâce à un engagement des hautes autorités de l´Etat et l´accompagnement des partenaires techniques et financiers, des efforts considérables ont été fournis ces dernières années dans le cadre de l´atteinte de l´objectif de développement durable N°3 en général et plus particulièrement celui en lien avec la santé des franges les plus vulnérables de la population à savoir les femmes, les enfants et les adolescents et jeunes (ODD N°3.1 et 3.22). Cependant depuis la déclaration du premier cas de la maladie à coronavirus, une baisse de l'utilisation des services est observée dans la plupart des structures sanitaires, ce qui risque d´anéantir les efforts fournis jusqu´ici et augmenter le nombre de décès extrahospitaliers de ces groupes liés aux autres maladies [2]. C'est pourquoi, conformément aux recommandations de l'Organisation Mondiale de la Santé, des mesures doivent être planifiées pour sauvegarder les activités essentielles de prévention et de soins destinés aux groupes les plus vulnérables dont les femmes, enfants et les jeunes. Notre étude a pour objectif d'évaluer les conséquences de la COVID-19 sur les activités curatives et préventives des formations sanitaires.

## Méthodes

**Type de l´étude, source de données et période de l´étude:** nous avons réalisé une étude rétrospective, descriptive des données sanitaires agrégées via le Système National d'Information sanitaire (DIHS2) et complétées au besoin à travers les registres de consultation externes et autres supports de statistique au niveau de district sanitaire. Nous avons inclus les données recueillies durant les deux premiers trimestres des années 2019 et 2020.

**Cadre de l´étude et approche de sélection de formations sanitaires et de participants:** ce travail a concerné dix-sept formations sanitaires publiques de premier niveau ou Centres de Santé Intégrés (CSI), reparties dans cinq Districts Sanitaires (DS) de la communauté urbaine de Niamey ; la ville capitale de Niger en Afrique de l´Ouest. Les centres de santés privés et non-intégrés dans le système de santé national ont été exclus. Les CSI ont été répartis comme suit: DS Niamey 1 (6 CSI), DS Niamey 2 (2 CSI), DS Niamey 3 (2 CSI), DS Niamey 4 (4 CSI), DS Niamey 5 (3 CSI). Seuls les patients vus en consultations externes ont été inclus dans l´étude.

**Analyses de données et considération éthique:** le paquet de prestations comprend les soins préventifs et curatifs à savoir la vaccination, la consultation prénatale, la consultation des nourrissons, l´accouchement assisté, etc. Pour évaluer les conséquences de la COVID-19 sur les activités curatives et préventives des formations sanitaires durant la période d´étude nous avons comparé les statistiques des consultations externes (curatives et préventives: consultation prénatale-CPN et vaccination des enfants au pentavalent première et troisième dose-Penta 1 et 3 et contre la varicelle-VAR 1) du premier semestre (janvier-juin) de l'année 2019 à celles de la même période de l´année 2020. Nous avons calculé les variations de proportion de taux d´utilisation de services curatifs, de la consultation prénatale et de la vaccination par le Penta 1 et 3 et par le VAR. Nous avons rapporté les moyennes et estimé les différences statistiques entre différents mois, trimestres et années par le test de t de student (moyennes), chi-carre (proportions) et ANNOVA (plusieurs moyennes) selon les cas. Une correction pour la multiplicité des tests a été réalisée par le test de Bonferroni. Le p-value <0.05 a été prise pour le degré de différence statistique. Les résultats ont été analysés à l'aide du graphPad Prism V.8 et de STATA V.15.

**Accord d´éthique et le consentement à participer:** cette étude a utilisé des données de programme de routine collectées rétrospectivement sans identificateurs de données individuels révélés ou utilisés. L´étude a été approuvée par le Niger, le ministère de la Santé publique et le Comité national de réponse multisectoriel COVID-19 du Niger.

## Résultats

**Figure 1:**
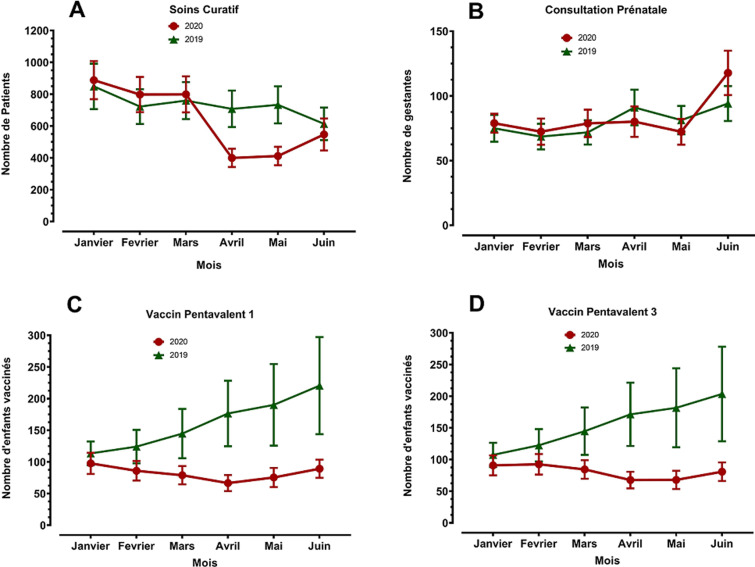
variation de l´utilisation de différents services de soins dans 17 centres de santé intégrés au Niamey en comparant la première moitié de l´année 2020 à celle de l´année 2019

La Figure 1 montre la variation de l´utilisation de différents services de soins dans 17 CSI au Niamey de manière générale en comparant la première moitié de l´année 2020 a celle de l´année 2019. Il y a eu une réduction moyenne pour les soins curatifs de 12% (95% intervalle de confiance-IC: -18% à -6%), pour la vaccination au pentavalent 1 de 49% (95%IC: -58% à -40%), pour la vaccination au pentavalent 3 de 48% (95%IC: -57% à -39%) et pour la VAR 1 de 35% (95%IC: -44% à -26%). Seule, une légère augmentation de 4% (95%IC: 0% à 8%) fut observée pour les consultations prénatales (CPN) grâce aux efforts réalisés au mois de juin et qui ont consolidé les acquis du premier trimestre. De manière particulière, les différences furent plus prononcées au deuxième trimestre 2020, avec une réduction de 34% (95%IC: -47% à -21%) pour les soins curatifs, de 61% (95%IC: -74% à -48%) pour la vaccination aux pentavalents 1 et 3 et de 36% (95%IC: -49% à -23%) pour la VAR. Un gain quasi nul de 1% (95%IC: -2% à 4%) fut noté pour la fréquentation à la CPN annulant ainsi les acquis du premier trimestre.

**Figure 2:**
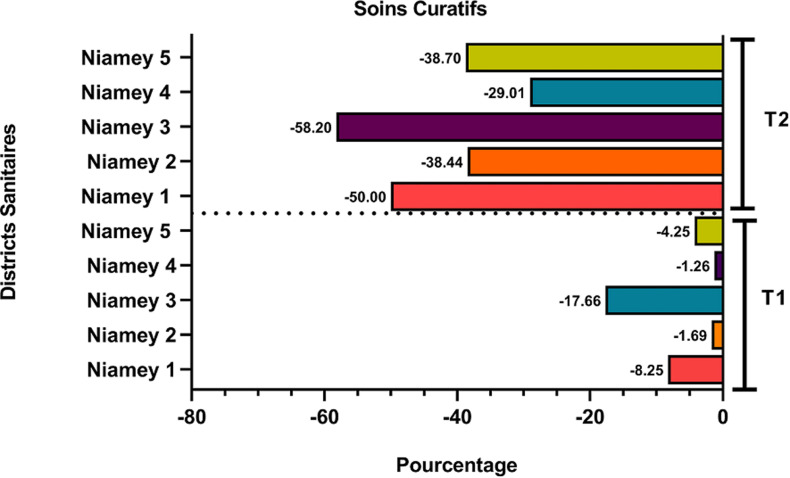
variation dans l´utilisation de services curatifs au Niamey au deuxième trimestre par district sanitaire en comparant la première moitié de l´année 2020 à celle de l´année 2019

Nous avons noté une baisse significative de l´utilisation des services curatifs au niveau de l´ensemble des DS au deuxième trimestre de l´année 2020. La baisse de la fréquentation des services a varié entre 29% à 58%. Le DS Niamey 3 a été affecté avec une baisse de l´utilisation des services curatifs de près de 60% au second trimestre 2020 comparé à la même période de 2019 (Figure 2). Contrairement à l´utilisation des services curatifs, seuls 10 CSI sur les 17 ciblés ont enregistré une baisse dans l´utilisation des services de consultation prénatale par les femmes enceintes, il s´agit essentiellement des CSI des DS Niamey 1, 2 et 3, avec une variation allant de 131 à 2191 femmes enceintes. Les 7 CSI des DS Niamey 4 et 5 ont pour leur part enregistré une hausse significative du nombre de femmes enceintes, cette hausse a atteint 30% pour le DS Niamey 4 au deuxième trimestre 2020. Les services d´immunisation ont également connu une baisse de leur utilisation. Après analyse, seul le DS Niamey 4 n´a pas enregistré de baisse de l´utilisation des services de vaccination pendant la période considérée. Les DS les plus affectés sont le DS Niamey 2 et Niamey 5 avec une baisse de plus de 3100 enfants non vaccinés au second trimestre de l´année 2020, soit un peu plus de 80%, comparativement à 2019. Les mêmes constats que précédemment sont fait pour l´immunisation avec la 3^e^ dose du pentavalent, les baisses les plus importantes ont été enregistrées au niveau des DS Niamey 2 et 5.

## Discussion

Cette analyse qui porte sur la comparaison de l´utilisation des services curatifs et préventifs sur une période de six mois au niveau de 17 CSI de la région de Niamey en période pré et per pandémique de la COVID-19 relève une réduction importante de la consommation des soins de santé primaire depuis l´annonce du premier cas de la COVID-19 au Niger intervenue le 19 mars 2020. Cette réduction est très substantielle surtout pour la vaccination des enfants au pentavalents.

Pendant que d´importants progrès furent observés au début du premier trimestre 2020 vs 2019 sur l´accroissement du taux de vaccination, il a été observé que plus de la moitié des enfants ont malheureusement raté leur dose de vaccination aux pentavalents 1 et 3. Le vaccin pentavalent qui comprend cinq vaccins en un protège les enfants contre la diphtérie, le tétanos, la coqueluche, l´hépatite B et contre *Haemophilus influenzae* type b (Hib), à l´origine de la pneumonie et de la méningite, cause majeure de décès et d´invalidité des enfants dans les pays à ressources limitées [3]. Tout en se focalisant sur les autres vaccins, les programmes élargis de vaccination devraient développer des stratégies de rattrapage pour ces vaccins ratés afin d´éviter les décès infantiles indirectement liés à la pandémie de la COVID-19, plus grave que la pandémie elle-même [4, 5]. En raison de l´accompagnement dont le CSI Saga a bénéficié de la part d´un de ses partenaires dans le cadre du renforcement de cette activité, à travers le financement des sorties foraines de vaccination pendant la gestion de la COVID-19, on note une faible variation dans le programme de l´immunisation à Niamey 4. Il est donc possible qu´avec l´appui des différents partenaires, le système existant du programme élargi de vaccination soit renforcé par des processus novateurs relevant de la science de l´implémentation [6].

Nos résultats sont en accord avec beaucoup d´autres pays sur la baisse de l´utilisation de service de soins curatifs en temps de la pandémie [2]. Une enquête de l´Organisation mondiale de la Santé [7] a révélé des perturbations des services de santé plus parmi les pays à faible revenu. Parmi les raisons évoquées, on note la peur d´être infecté durant la visite d´un établissement de soins, l´incapacité d´accéder aux soins en raison des politiques de confinement, la suspension et l´annulation de services comme la chirurgie élective et la stigmatisation qui accompagne un malade tousseur qui est directement affiliée au patient COVID-19 et la peur de la mise en quarantaine. La baisse de l´utilisation des soins curatifs au niveau primaire s´accompagnera d´une mortalité à domicile pour beaucoup de maladies comme l´infarctus du myocarde et le déséquilibre des maladies chroniques comme le diabète, l´hypertension et le VIH et le sous rapportage et la propagation des maladies transmissibles comme la tuberculose pulmonaire ainsi que le recours à l´automédication avec risque de surdosage et intoxication aux produits indigènes [2, 7].

La réduction des interventions essentielles en matière de santé maternelle durant la période de la pandémie a été observée dans beaucoup de pays [8, 9]. Si la femme enceinte est permanemment en questionnement sur l´issue de la grossesse et le bien-être de son enfant, la pandémie a infligé un stress de plus avec un état d´anxiété et de dépression à celui préexistant [10]. A défaut de la CPN, le risque de décès materno-infantile s´accroit ainsi que le risque de toutes les autres morbidités relatives à l´état gravidique (avortement, prématurité, accouchement à domicile avec ses risques comme l´hémorragie du postpartum, etc.), perturbant ainsi les acquis de développement durable. Offrir des services de CPN devient une urgence de santé publique en temps de la pandémie. Des questions persistantes cependant avec la possibilité d´une transmission verticale de l´infection à COVID-19 et de la grossesse comme facteur de risque de mortalité due à la COVID-19 (surtout de l´hospitalisation) demeurent des éléments importants à considérer. La télémédecine par exemple en Chine a diminué les problèmes psychologiques chez les femmes enceintes [10]. Si ces genres de solution peuvent être possibles dans certains endroits en Afrique, il appert que dans beaucoup de CSI, rien de tel ne peut être organisé. Cependant, des mécanismes adaptatifs comme pour la tuberculose et le VIH avec l´utilisation des communautaires, femmes sages et cliniques mobiles peuvent être mis en place dans des pays à faible revenue.

## Conclusion

La pandémie de la COVID-19 impacte négativement sur les prestations de service destinées aux groupes les plus à risques de la population à savoir les femmes et les enfants. Pour une plus grande résilience de structures de soins, des efforts supplémentaires doivent être fournis par les gouvernements et leurs partenaires pour: i) une amélioration de la qualité des services de soins; ii) la mise en place des moyens suffisants de prévention contre la COVID-19 au niveau des formations sanitaires et le renforcement de la sensibilisation afin redonner confiance aux utilisateurs; et iii) la mobilisation des ressources supplémentaires pour la mise en place des plans de continuité des services pendant cette période.

### Etat des connaissances sur le sujet

- La pandémie de COVID-19 a provoqué une crise sanitaire en Afrique comme au Niger et continue de faire des ravages dans le monde entier;
- L´impact de cette pandémie sur l´utilisation de soins curatifs et préventifs en Afrique comme au Niger n´est pas encore bien connue en ce jour.

### Contribution de notre étude à la connaissance

- La pandémie de COVID-19 a eu un effet négatif significatif sur l´utilisation des services de santé au Niger;
- La pandémie de la COVID-19 a impacté surtout les prestations de service destinées aux groupes les plus à risques de la population à savoir les femmes (consultations prénatales) et les enfants (vaccinations).
